# The silver effect of admission glucose level on excellent outcome in thrombolysed stroke patients

**DOI:** 10.1007/s00415-018-8896-6

**Published:** 2018-05-18

**Authors:** Charlotte Rosso, Flore Baronnet, Belen Diaz, Raphael Le Bouc, Giulia Frasca Polara, Eric Jr Moulton, Sandrine Deltour, Anne Leger, Sophie Crozier, Yves Samson

**Affiliations:** 1 Inserm U 1127, CNRS UMR 7225, UMR S 1127, Institut du Cerveau et de la Moelle épinière, ICM, Sorbonne Université, 75013 Paris, France; 2 APHP, Urgences Cérébro-Vasculaires, Hôpital Pitié-Salpêtrière, 47-83 Boulevard de l’Hôpital, 75013 Paris, France

**Keywords:** Acute stroke, Hyperglycemia, Cohort studies, Prognosis

## Abstract

Higher admission glucose levels (AGL) are associated with less favorable outcome in thrombolysis. But, could AGL’s impact on outcome vary by onset-to-treatment (OTT) time? Is hyperglycemia associated with a shorter therapeutic time window for excellent outcome for thrombolysed stroke patients? We assessed predictive values of AGL, baseline NIHSS, age, and OTT time quartiles on excellent outcome (3-month modified Rankin score of 0–1) in 773 patients treated by rt-Pa. We added the AGL × OTT time quartile interaction in the model and separately analyzed the predictive values of AGL, age, and NIHSS for each OTT time quartile if the interaction was significant. AGL, baseline NIHSS, age, and OTT time quartiles were significant predictors. When added in the model, the AGL × OTT interaction was significant (OR: 0.96, 95% CI: 0.94–0.99, *p*: 0.0009). AGL was predictive only during the third OTT time quartile (181–224 min). During this period, the predicted rate of excellent outcome was 16% for AGL = 6.5 mmol/L and 8% for AGL = 8 mmol/L. The rate of excellent outcome was not decreased in hyperglycemic patients for OTT time ≤ 180 min (20 vs. 24.5% *p*: 0.37), but was decreased for OTT time > 180 min (9.6 vs. 26.7% *p*: 0.00001). Similar results were found in patients with MCA recanalization, but not in patients without recanalization. The therapeutic time window for excellent outcome is shortened in hyperglycemic patients. This would support the design of “freezing penumbra” randomized trials based on ultra-early AGL control.

## Introduction

In the era of recanalization therapies, the factors responsible for the onset-to-treatment (OTT) time vs. outcome relationship need further investigations in acute stroke patients. Treating the factors that shorten the therapeutic time window for excellent outcome in fact may correspond to slow down the penumbra’s evolution. Baseline post-stroke hyperglycemia could be one of these factors. To date, most studies showed that higher admission glucose levels (AGL) were associated with less favorable outcome in thrombolysis [1], but could AGL’s impact on outcome vary by OTT time?

This possibility arises from an association between hyperglycemia and an accelerated infarct growth in experimental and imaging studies as reviewed in Piironen et al. [2]. Moreover, in these studies, there is accumulating evidence indicating that the glucose toxicity threshold was low (between 6 and 8 mmol/l) [3]. To our knowledge, the interaction between OTT time and AGL has never been formally tested.

**Fig. 1:**
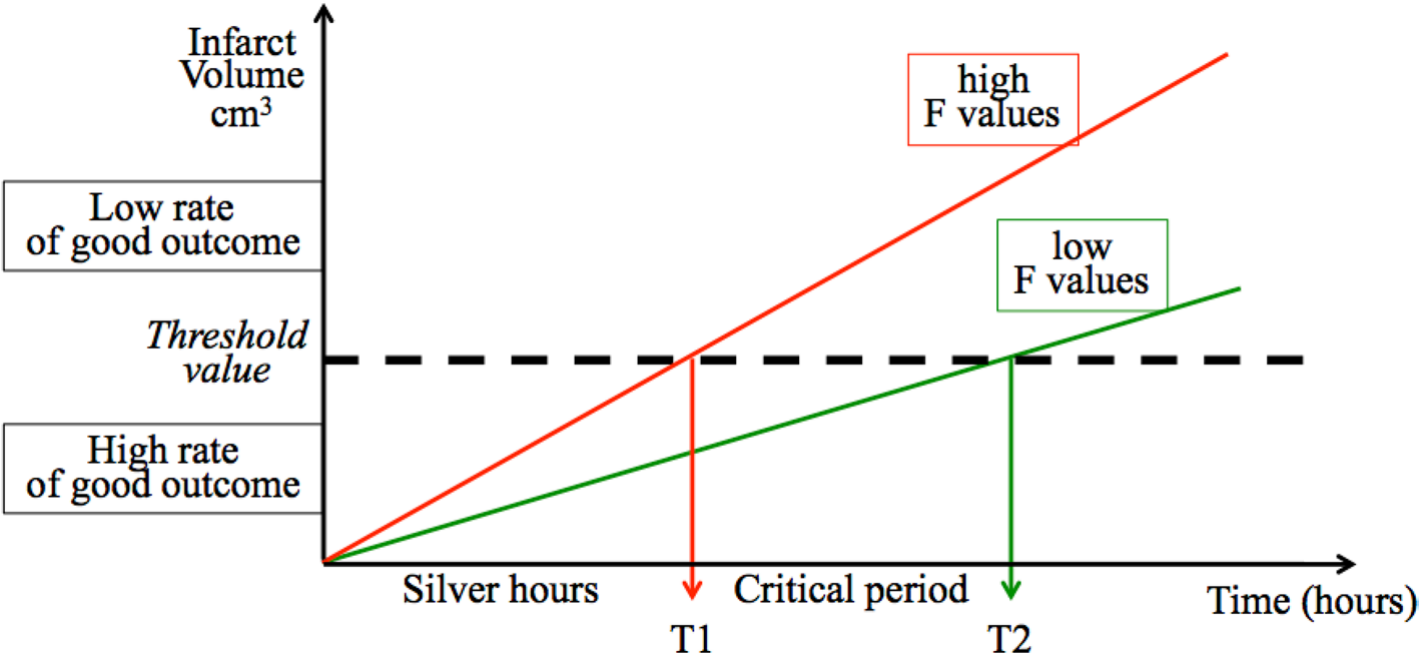
The silver effect

The proportion of patients with excellent outcome after thrombolysis sharply decreases when a threshold volume of infarction is reached, corresponding to a certain amount of penumbra volume being transformed. Parsons et al. reported that 48% of patients with diffusion-weighted imaging (DWI) lesions < 18 ml at admission had excellent outcome at 3 months, compared to only 15% with DWI lesions > 18 ml [4], with similar results reported by others [5–7]. Our hypothesis is that the true effect of OTT time on infarct volume may actually be changed by a penumbra-modifying factor, such as AGL (Fig. 1). More precisely and theoretically (Fig. 1), if a factor *F* (i.e., a penumbra-modifying factor, such as AGL) increases the rate of infarct growth, the infarct volume threshold for excellent outcome will be reached at an earlier time (T1) for higher values of *F*. Conversely, lower values of F can extend this cut-off point to a later time (T2). Prior to T1, the rate of excellent outcome will be high for any value of *F*, and *F* will not statistically predict outcome. We called this period (before T1) the “silver hours”, by analogy with the golden hour [8]. During the subsequent “critical period” (T1–T2), the rate of excellent outcome will decrease faster for higher values of *F*, which will ultimately predict outcome. Beyond T2 or in the absence of recanalization, the rate of excellent outcome will continue to decrease even for low values of *F*, which will lose its predictive value. In other words, a “silver effect” predicts a significant interaction between OTT time and *F*, with the predictive value of *F* for excellent outcome being low during the silver hours, high during the critical T1–T2 period, and low again beyond T2. A “silver effect” also implies that the OTT time × *F* interaction should be significant in patients with successful thrombolysis but not in those with persistent occlusion. In this latter, time goes on beyond T2 and, whatever the level of *F*, patients are more likely to have a poor outcome as the infarct volume will grow up to the critical threshold.

We investigated this issue in our prospective registry of patients treated by intravenous thrombolysis. We hypothesized that AGL at admission could constitute a hypothetical factor *F* and tested the interaction between AGL and OTT time on outcome.

## Methods

### Patients

We used the data of the Pitie-Salpêtrière prospective registry of patients treated by intravenous rtPA for an anterior circulation ischemic stroke. Between February 2000 and February 2016, 904 consecutive patients were treated. According to the routine clinical protocol at our institution, we treated the patients within a 4.5-h time window with the usual dose (0.9 mg/kg for a maximal dose of 90 mg). Symptom onset was defined as the last time period that the patient had exhibited normal health. Admission serum glucose level was measured before any treatment and available in 890 patients (98.5%) in the registry. A neurological examination was assessed using the National Institute of Health Stroke Scale (NIHSS) at admission (prior to the magnetic resonance imaging-MRI and any treatment) and on days one (D1) and seven (D7). Modified Rankin scores (mRs) were assessed at 3 months in routine and available in the registry for 773 patients (86.9%).

### Data analysis

Descriptive statistics consisted of median and interquartile ranges (IQR). Comparisons of proportions were determined by a Chi-squared test.

#### Impact of AGL and OTT time on excellent outcome

We used the 3-month mRs of 0 or 1 as a primary outcome criterion for excellent outcome. Since NIHSS, age, OTT time, and AGL are robust predictors of outcome in most studies, we first investigated their univariate and then their multivariate predictive values. OTT time was divided into quartiles. The multivariate analysis was done using stepwise logistic regression, the variables being entered in the final model at *p* ≤ 0.05 and removed at *p* > 0.10.

To test the “silver effect” hypothesis, we then added in the model the OTT time quartile × AGL interaction. In the case of a significant interaction, we planned to run stepwise logistic regressions in the four OTT time quartiles using age, NIHSS, and AGL as independent variables. When AGL was a significant predictor, we calculated the best sensitivity-specificity threshold of AGL associated with excellent outcome using ROC curve analysis.

As stated in the introduction, a “silver effect” also implies that the OTT time × AGL interaction should be significant in patients with successful thrombolysis (i.e., recanalization) but not in those with persistent occlusion. We therefore compared the results in subgroups of patients with and without complete middle cerebral artery recanalization. Intracranial occlusion and arterial recanalization were assessed on initial (< 6 h) and follow-up MR interpretable angiography (at 24 h), respectively (*n* = 724, 94% and *n* = 658, 85%). Recanalization was considered on a 3-item scale: (1) patent or complete recanalization; (2) partial or minimal flow-related signal in the region of the arterial clot; and (3) persistent occlusion.

#### Impact of AGL and OTT time on good outcome

The analyses were additionally performed on 3-month mRs scores of 0–2 to verify that good outcome is also potentially affected by a “silver effect”.

## Results

### Patients

Among the 773 included patients, 52.5% (*n* = 406) were men. Thirty-six patients were additionally treated by endovascular treatment (4.6%). The median and interquartile (IQR) values of admission NIHSS was 16 (11–21), age: 67 years (53–80 years), AGL: 6.5 (5.8–7.8) mmol/L, and OTT time: 181 min (142–225 min). The 131 patients excluded for missing data were comparable to those included for baseline NIHSS (median NIHSS: 16 vs. 16, *p*: 0.44) but were older than the included ones (median age: 74 vs. 67 years, *p*: 0.002). Day-one median NIHSS was 12 (IQR: 7–19) and day-seven NIHSS was 8 (IQR: 2–13). At 3 months, excellent outcome (mRs 0–1) was achieved in 20.2% (*n* = 156) of patients, good outcome (mRs 0–2) in 40.2% (*n* = 311), and the death rate was 17.1% (*n* = 132).

At admission, intracranial MRA revealed MCA trunk (M1) occlusions in 17% (*n* = 125) of patients, MCA branches (M2) occlusions in 40% (*n* = 292), intracranial occlusions of the carotid artery in 29% (*n* = 211), miscellaneous occlusions in 4.5% (*n* = 34), and no occlusions in 9.5% (*n* = 62). At 24 h, the MCA was patent in 46.6% (*n* = 306) of cases, partially recanalized in 24% (*n* = 158), and remained occluded in 29.4% (*n* = 194). Symptomatic hemorrhage occurred in 7.5% (58/765).

The characteristics of the patients sorted in the four OTT time quartiles (< 142 min; 142–180; 181–224; > 224 min) are shown in Table 1. The four groups were similar except that patients in the first quartile were older than those of the fourth quartile (*p*: 0.002). The rate of excellent outcome decreased significantly from 25.5% in the first OTT time quartile, to 16.4% in the last OTT time quartile (*p*: 0.03).

**Table 1:** Characteristics of the patients in the four OTT quartiles

OTT quartiles	OTT time (min)	Baseline NIHSS	AGL (mmol/l)	Age (years)	mRs 0–1 (%)
1st; <142 min (n = 193)	120 (109–139)	16 (11–22)	6.5 (5.8–7.7)	70.5 (55.7–81.9)	25.5
2nd; 142–180 min (n = 192)	163 (152–175)	17 (11–22)	6.5 (5.8–8)	66.9 (53.8–81.3)	19.7
3rd; 181–224 min (n = 193)	200 (190–210)	16 (11–21)	6.5 (5.7–7.7)	66.3 (53.9–80)	19.7
4th; >224 min (n = 195)	260 (240–284)	15 (10–20)	6.6 (5.8–8.3)	63.2 (49.5–77.2)	16.4

Values are median and IQR

### Prediction of excellent outcome (mRs 0–1)

NIHSS, age, OTT time quartile, and AGL were significant predictors in the univariate analysis and remained significant in the multivariate stepwise logistic regression (Table 2). When the OTT time quartile*AGL interaction was introduced in the logistic regression, the final model retained three independent predictors: NIHSS (OR: 0.84, 95% CI: 0.81–0.87, *p* < 0.00001), age (OR: 0.98, 95% CI: 0.97–0.99, *p* = 0.002) and the OTT time quartile*AGL interaction (OR: 0.96, 95% CI: 0.94–0.99, *p* = 0.0009).

**Table 2:** Predictive values of AGL, OTT time quartiles, baseline NIHSS, and age on 3 months excellent outcome (mRS 0–1)

	Univariate analysis		Multivariate analysis	
	OR, 95% CI	*p*	OR, 95% CI	*p*
AGL (mmol/l)	0.84 (0.76–0.93)	0.001	0.89 (0.80–0.98)	0.02
OTT time quartile	0.85 (0.72–0.99)	0.04	0.77 (0.65–0.92)	0.004
Baseline NIHSS	0.84 (0.81–0.87)	0.00001	0.84 (0.81–0.87)	0.00001
Age	0.98 (0.97–0.99)	0.0002	0.98 (0.97–0.99)	0.001

The multivariate analysis is the final model of the stepwise logistic regression when the four variables were entered without entering the AGL × OTT time quartile interaction term

Since the interaction term was significant, we analyzed the predictive values of AGL in the four OTT time quartiles. AGL was a significant predictor only in the third OTT time quartile (181–224 min), with a 52% decreased probability of excellent outcome per mmol/L increase (OR: 0.48; 95% CI: 0.33–0.70, *p* < 0.0001). The ROC curve analysis of AGL predicting 3-month excellent outcome was also significant only in the third OTT time quartile. The AUC was 0.73 (95% CI: 0.67–0.79, *p*: 0.0001), corresponding to good diagnostic performance [9]. The best compromise between sensitivity and specificity to predict excellent outcome was for a threshold of AGL ≤ 6.5 mmol/l, yielding a sensitivity of 81.6% (95% CI: 65.7–92.2) and a specificity of 56.8% (95% CI: 48.6–64.7). The low specificity, however, indicated that the false positive rate was high (patients with low AGL and poor outcome).

The final multivariate logistic regression models are shown in Table 3. These models predicted a rate of excellent outcome for a “median” patient (NIHSS = 16, 67 years old, AGL = 6.5 mmol/L (118 mg/dL)) of 26% during the first quartile (OTT time < 142 min), 16% during the second and third quartile, and 10% during the last quartile (OTT time > 224 min). AGL was an independent predictor only during the third quartile (181–224 min). During this “critical period”, the predicted rate of excellent outcome decreased from 16% for AGL = 6.5 mmol/l (118 mg/dL) to 8% for 8 mmol/l (145 mg/dL).

**Table 3:** Final model of the multivariate logistic regression in the four OTT quartiles for excellent outcome

OTT time quartiles	< 142 min	142–180 min	181–224 min	> 224 min
AGL	–	–	0.57 (0.38–0.86) *p* = 0.008	–
NIHSS	0.84 (0.79–0.90) *p* = 0.00001	0.86 (0.81–0.92) *p* = 0.00001	0.83 (0.77–0.90) *p* = 0.00001	0.83 (0.77–0.90) *p* = 0.00001
Age	–	0.96 (0.94–0.99) *p* = 0.002	–	0.97 (0.95–0.998) *p* = 0.04

As expected, the rate of excellent outcome differed in patients with and without complete MCA recanalization (32.9 vs. 11.9%, *p* < 0.0001). In patients with MCA recanalization, the multivariate logistic regression retained the same variables as in the complete analysis: OTT time quartiles*AGL interaction (OR: 0.96; 95% CI: 0.93–0.98, *p* = 0.001), age (OR: 0.98; 95% CI: 0.96–0.99, *p* = 0.004), and NIHSS (OR: 0.89; 95% CI: 0.85–0.93, *p* < 0.00001). Similar to the complete analysis, the logistic regressions performed with the different quartiles revealed that AGL was a significant predictor only in the third OTT time quartile (OR: 0.44; 95% CI: 0.27–0.73, *p* = 0.001), whereas NIHSS was a significant predictor in the three other quartiles.

In patients without complete recanalization, the multivariate logistic regression retained the baseline NIHSS as a predictor of excellent outcome (OR: 0.78; 95% CI: 0.73–0.84, *p* < 0.00001), and hyperglycemia did not affect the rate of excellent outcome.

### Prediction of good outcome (mRs 0–2)

The multivariate prediction of 3 months good outcome (mRs 0–2) gave similar results to the prediction of excellent outcome with 3 independent predictors: OTT time*AGL interaction (OR: 0.97, 95% CI: 0.95–0.98; *p* = 0.0001), age (OR: 0.96, 95% CI: 0.95–0.98; *p* < 0.00001), and NIHSS (OR: 0.83, 95% CI: 0.80–0.85; *p* < 0.00001).

## Discussion

We found that AGL was predictor of excellent and good outcome even after adjustment for NIHSS, age, and OTT time. The results were highly consistent with those of a recent systematic review [1]. The new finding was the existence of a strong interaction between AGL and the OTT time quartile. This indicates that the relation between AGL and excellent outcome varies with OTT time. Further analyses suggested that hyperglycemia was associated with a shorter therapeutic time window for excellent outcome with what we called a “silver effect”. In subgroup analyses, these findings were only observed in the case of complete MCA recanalization.

### Hyperglycemia decreases the time window for excellent outcome after thrombolysis

AGL was a significant predictor only during the third quartile (181–224 min). In this critical period, the predicted rate of excellent outcome decreased from 16 to 8% when, for example, AGL increased from 6.5 to 8 mmol/L (118 to 145 mg/dL). A cutoff AGL of 6.5 mmol/L was identified by the ROC curve analysis, close to the 6.8 mmol/L (123 mg/dL) value reported for good outcome in the SITS-ISTR register [10]. Using the 6.5 mmol/l cutoff, the rate of excellent outcome was not affected by hyperglycemia when OTT time was ≤ 3 h but was markedly decreased by hyperglycemia when OTT time was between 3 and 3 h 44 min. This is consistent with the report of Ribo et al. who found that the time to arterial recanalization leading to poor outcome (mRs > 2) was much shorter (3.5 h) in patients with hyperglycemia than in patients with adequate glucose control (7.5 h). Moreover, the authors report that, on average, the infarct grew 2.7 times faster in patients with hyperglycemia during arterial occlusion [11]. This is perhaps why ultrasound-enhanced thrombolysis, which speeds up arterial recanalization [12], mitigated the detrimental effect of increasing AGL on good outcome in the CLOTBUST trial [13].

### The “silver effect” of AGL

The “silver effect” implies that the relation between AGL and excellent outcome would be maximal during a critical period of OTT time (between T1 and T2 on Fig. 1), and this should selectively be observed in patients with successful thrombolysis. Our results provide evidence for the existence of a silver effect since the AGL×OTT time quartile interaction was significant, the negative relation between AGL and outcome was observed only the third OTT quartile, and these findings were selectively found in patients with complete MCA recanalization. The link between the predictive value of AGL on outcome and time to early recanalization has been previously reported by Alvarez-Sabin et al. [14, 15]. However, in these studies, AGL was a predictor even if recanalization occurred ≤ 180 min, whereas in our study, AGL predicted outcome only in the 181–224 min OTT time period. The discrepancy may have several explanations. First, the 180 min “silver hours” in our study is based on the arbitrary division of OTT time in quartiles. Second, Fig. 1 shows that the timing of T1 and T2 will depend on the rate of infarct growth, the impact of AGL on infarct growth, and the value of the infarct threshold volume. Since these three parameters are variable, the exact timing of the critical period may differ between studies and may have been shorter in studies by Alvarez-Sabin et al. [14, 15]. This is also consistent with the results of previous MRI-based studies [8, 16–18, 16–18].

### General comments and limitations

Our results indicated that glucose control is unlikely to improve the rate of excellent outcome unless the treatment started less than 2–3 h after stroke onset. This may in part explain the negative results of published insulin trials, which generally included patients < 24 h post-stroke [19]. Even in INSULINFARCT, which included patients < 6 h post-stroke onset, only 22 patients were treated within 2 h [20]. The authors also suggested that strict glucose level control, perhaps even above 6.5 mmol/l, should be targeted.

Our results should not be over-interpreted. First, the shorter time window for excellent outcome did not prove that the time window for thrombolysis is shorter in hyperglycemic patients. This would require the demonstration of an AGL × OTT time × treatment interaction in a study with control subjects. Of note, in the IST trial, which randomized about three-quarters of the patients after 3 h, the AGL × treatment interaction was not significant, but AGL values spanning 5–8 mmol/L were merged in the same category [21]. Second, the statistical associations described here do not formally prove a causal link between AGL and outcome. This will remain uncertain unless “freezing” penumbra trials turn out to be positive. Third, the “silver effect” itself is an appealing hypothesis, but remains as such since OTT time is an imprecise surrogate marker of infarct growth [22]. Furthermore, the timing of recanalization was unknown, we cannot exclude that it was slowed down. Therefore, it would be of interest to investigate in patients treated with EVT whether there is a significant AGL × time to reperfusion interaction and if the steep declines in rates of excellent and good outcome with onset to reperfusion times between 190 and 390 min (efig 5 in Saver et al. [6]) are modified by AGL. This would be an important argument in favor of “freezing the penumbra” therapeutic strategies by ultra-early glycemic control. One experimental study suggests that this approach may be beneficial [23]. Yet, this remains to be proven by randomized trials, since the pathophysiological mechanisms linking hyperglycemia and acute ischemia are complex and still debated [24], and intensive insulin treatment may paradoxically increase infarct growth [20, 25].

## Conclusions

In this study, we highlighted that the relation between AGL and excellent/good outcome varies by OTT time. In other words, hyperglycemia was associated with a shorter therapeutic time window for excellent/good outcome with what we called a “silver effect”. These results would support the design of “freezing penumbra” randomized trials based on ultra-early AGL control (< 3 h) targeting nearly normal glycemic levels (< 6.5 mmol/L, i.e., 115 mg/dL).
